# Correlation between Campus-Built Environment and Physical Fitness in College Students in Xi’an—A GIS Approach

**DOI:** 10.3390/ijerph19137948

**Published:** 2022-06-29

**Authors:** Zijun Lu, Zhengao Li, Chuangui Mao, Yuanyuan Tan, Xingyue Zhang, Ling Zhang, Wenfei Zhu, Yuliang Sun

**Affiliations:** Department of Exercise Science, School of Physical Education, Shaanxi Normal University, Xi’an 710119, China; luzijun@snnu.edu.cn (Z.L.); lizhengao@snnu.edu.cn (Z.L.); maocg117@snnu.edu.cn (C.M.); tanyy98@yeah.net (Y.T.); zhangxingyue@snnu.edu.cn (X.Z.); zhangling1@snnu.edu.cn (L.Z.)

**Keywords:** built environment, physical fitness, GIS

## Abstract

Background: This research aimed to investigate the correlation between students’ physical fitness and campus-built environment, which could put forward some suggestions for the construction of a campus environment. Method: Four colleges in Xi’an were regarded as special “semi-closed” spaces. Combined with ArcGIS and SPSS, the correlation between the built environment of colleges and the students’ physical fitness test results in 2019 was analyzed (*n* = 1498). Results: regarding the men questioned in this research, there was a significant correlation between street connectivity and vital capacity, grip strength, 50 m running, 1000 m running, a significant correlation between land use mix and vital capacity, sit-and-reach, pull-up, grip strength, a significant correlation between green space per capita and vital capacity, grip strength, 50 m running, and a significant correlation between walk score and vital capacity, pull-up, grip strength, and 50 m running. Regarding the women questioned in this research, there was a significant correlation between street connectivity and vital capacity, grip strength, 50 m running, 800 m running, curl-up, a significant correlation between land use mix and vital capacity, sit-and-reach, curl-up, grip strength, 800 m running, a significant correlation between green space per capita and vital capacity, grip strength, curl-up, sit-and-reach, and a significant correlation between walk score and vital capacity, curl-up, grip strength, and 800 m running. Conclusion: the built environment on campus can indirectly affect the physical fitness of college students. Increasing the number of intersections and short connections of campus streets, ensuring that the green space of the campus meets the standards, and reasonably arranging the site selection of buildings are conducive to improving the physical fitness of students.

## 1. Introduction

The built environment is the spatial response of urban development and construction, which is closely related to the physical fitness of residents. The earliest research on the relationship between the built environment and physical activity was the “3D” model proposed by Cervero, which believes that density, diversity, and design will affect people’s travel times and route choice. Subsequently, destination accessibility and distance to transit are added to form the “5D” model [1]. Choi et al. summarized the personal factors and environmental factors associated with physical activity (90 personal factors and 27 environmental factors), and the results indicated that accessibility, convenience, and aesthetic had a significant positive correlation with physical activities and the physical fitness of residents [2]. When time was available, walking was the most popular method of exercising among healthy adults, and the density of fitness facilities and the green space per capita were significantly positively correlated with physical fitness [3]. Urban green areas and structures have a positive effect on people, which is why green spaces have to be connected and accessible. For example, residents could reach a green space from their homes in 10 min or from anywhere in the city in 15 min. It is worth mentioning that a recent study published in NATURE proved that built environment also has a positive effect on the cognitive function of humans [4].

Several studies in this area suggested that physical activities play an important role in the promotion of physical fitness, which could improve health and prevent various physical and mental illnesses [5]. A five-year study on physical fitness level and mortality in the United States showed that once people abandon the sedentary lifestyle and have “ordinary” physical fitness levels, they can significantly reduce mortality by 44% [6]. Previous studies have proved that the urban structure affected the community, and a collective embedding framework was used to study the effect, including the convenience of traffic, the safety of the community, the area of green space, the connection between the industrial land(or residential land) and roads [7,8,9]. On the contrary, an urban structure which was not well designed (the function of land use is simple, the population density is low, and the residential areas are mostly located in the peripheral areas of the city) would have a negative effect on physical fitness as the residents mainly used motorized transportation, which will reduce the frequency of daily physical activities [10].

However, most current studies focused on the correlation between the built environment and physical activities [2,11]. A few studies used physical activities as mediating variables to analyze the correlation between the built environment and physical fitness. Vanhelst et al. found that busy traffic conditions were significantly negatively associated with adolescent physical fitness [12]. On the contrary, a safe trail or bicycle path and well-designed exercise areas were effective in promoting adolescent physical fitness. Cheah et al. also demonstrated a significant correlation between nine environmental factors (street connectivity, land use mix, etc.) and aerobic capacity [1]. In addition, these studies mainly took interviews and questionnaires (e.g., NEWS, MAPS, etc.) as evaluation of the built environment [13,14,15,16,17,18,19]. The rate feedback of the results is highly volatile, which was vulnerable to subjective influences of the investigator and participants [20]. Geographic Information System (GIS) has been used in the measurement of the built environment as a spatial information analysis technology, which could effectively save time and obtain more objective data [21,22].

Most related research took residential communities and urban administrative districts as a buffer, while there was only small percentage of research on colleges. In consideration of high academic pressure, Chinese students’ rest and exercise time have been greatly compressed, and this situation continued until they entered the college. After that, students have more “free time”, and the campus becomes the main area for students to take physical activities. It should be noted that college students are in a special period (late adolescence and early adulthood), and the attitudes towards and knowledge of health formed at this time may continue throughout their lifespan.

Given the above, it is necessary to study the built environment of campus and students’ physical health. This study used the ArcMap component of ArcGIS system to calibrate the built environment of four colleges and calculate the corresponding data. ArcMap is a part of ArcGIS system which is developed by ESRI company whose main function is to process map information, edit GIS data, and process data automatically. Compared with questionnaires (NEWS, MAPS, etc.), the data obtained by ArcGIS system is not only more objective but also easier to analyze [22]. SPSS was finally used to analyze the correlation between the physical health of college students and the built environment on campus. Based on previous studies on green space and street connectivity, we hypothesize that there was a certain correlation between campus green space, street connectivity, and students’ physical fitness.

## 2. Methods

### 2.1. Participants

The objects of this study are students from four colleges in Xi’an, Shaanxi, who participated in the national student physique test in 2019 (Shaanxi Normal University (Chang’an campus) (S1), Xi’an University of Architecture and Technology (Cao tang campus) (S2), Xi’an Jiao tong University (Xing Qing campus) (S3), and Northwest University of Technology (Chang’an campus) (S4)). All the selected students completed all the test items of the physical fitness test whose birth dates are between 1997 and 2000 (Table 1) (*n* = 1498).

### 2.2. Measures of the Built Environment

**Figure 1:**
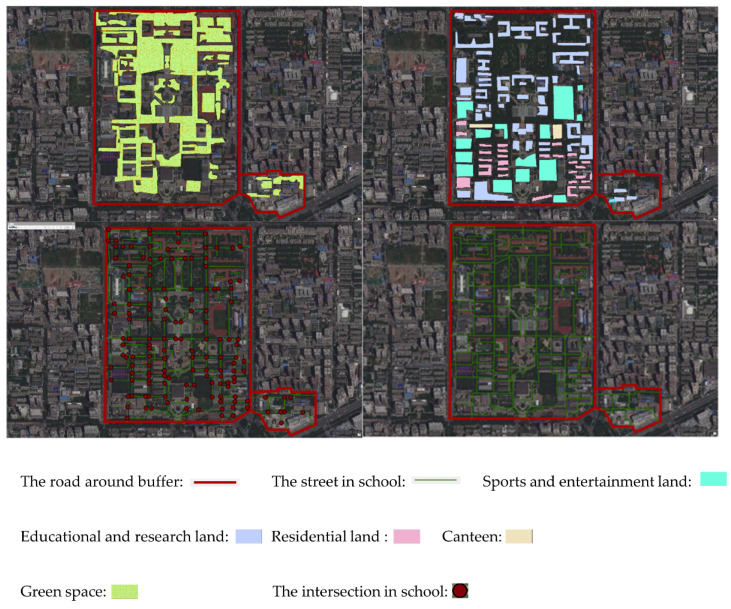
The ArcGis-processed image of one of the colleges.

Considering that there is no clear built environment index reference system in China, this research takes the built environment indexes related to “feasibility, comfort, and pleasure” in the reference system established by Alfonzo Ma as an example, and selects the following four indexes: street connectivity, land use mix, green space per capita, and walk score [23]. This study refers to Jason G. Su’s research which uses satellite data to study the correlation between green space in the buffer zone and the health of residents [24]. ArcGIS was used to build a buffer with roads around the four colleges, in which streets, intersections, and green spaces etched inside the campus are labeled (Figure 1).

#### 2.2.1. Calculation Method of Street Connectivity

**Figure 2:**
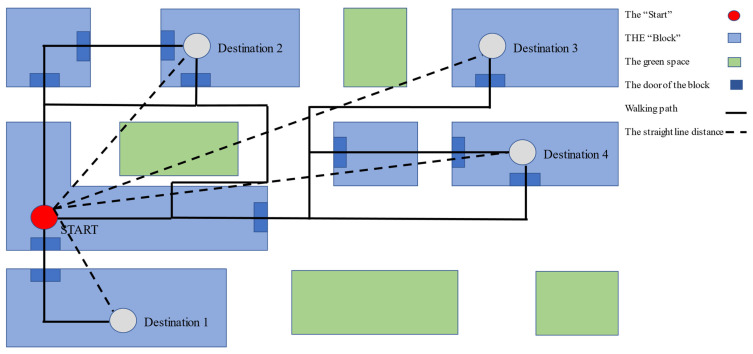
Mathematical Model of Street Connectivity Measurement.

Street connectivity (SC) is a measure of the density of network connections and the directness of paths. The better the street connectivity, the more the short links and intersections, which means people have more paths to destinations to choose [25] (Figure 2).

(1) $$\mathrm{Basic}\;\sin\mathrm{gle}-\mathrm{point}\;\mathrm{street}\;\mathrm{connectivity}:\;Q_{s}=\;\sum_{i=1}^{n=6}\left(a_{1}\times b_{1}÷c_{1}\right)$$

(2) $$\mathrm{Basic}\;\mathrm{area}\;\mathrm{street}\;\mathrm{connectivity}:\;Q_{a}=\;\sum_{i=1}^{n=6}\left(Q_{s}\times d_{1}\right)$$

*a*: walking distance: straight-line distance

*b*: the importance of walking destination (Table 2)

*c*: distance attenuation value

(3) $$C\left(y\right)=\{\begin{array}{c} 1\;;0<y<\frac{1}{2}\\ -\frac{5}{6}y+\frac{17}{12}\;;\;\frac{1}{2}<y<\frac{17}{10}\\ 0\;;y>\frac{17}{10} \end{array}$$

*C*(*y*): distance attenuation value; *y*: distance

*d*: the weight value

**Table 2:** Facility classification and weight table.

Facility Classification		Classification Weight	Total Weight
**Food and beverages**	Restaurant	0.75, 0.45, 0.25, 0.25, 0.2, 0.2, 0.15, 0.25	2.5
	Beverage shop	0.45, 0.2, 0.1	0.75
**Shopping**	Store	0.5, 0.25, 0.25	1
	Supermarket	0.5	0.5
**Leisure sport**	Library	0.5	0.5
	Park	1	1
	Fitness place	0.5, 0.25, 0.25	1
**Medical treatment**	Hospitals and pharmacies	1	1

#### 2.2.2. Calculation Method of Land Use Mix

Land use mix (LUM) is a popular concept in urban planning due to its expected role in improving environmental sustainability as well as the quality of citizen’s life. The current definition of land use mix mainly covered three dimensions, namely, distance, quantity, and “the attribute level”.

The evaluation index of land use mix degrees around the world is complex. The one used in this research comes from Frank, which measures the number of functional land use types and the ratio of area to total value [26]. Based on Chang Xia’s research on the land use mix in five major cities in China, the land use types are divided into residential land, educational and research land, sports and entertainment venues, and dining halls [27].

(4) $$LUM=\overset{n}{\underset{i=1}{\sum }}b_{i}\ln b_{i}÷\ln n$$

*LUM*: Land use mix;

*b*: Functional land in the buffer zone as a percentage of the total land area;

*n*: Number of lands used

#### 2.2.3. Calculation Method of Green Space per Capita in School

Green space per capita (GSPC) refers to the space created by plants, including green space or parks composed of lawn, shrubs, and trees, as well as indoor space built by plants or potted plants [28]. According to the attribute information for the “purpose”, green space could be divided into usable green space (urban parkland, indigenous forest, etc.) and non-usable green space (Farmland, commercial jungle, etc.) [29].

The latest research found that “per capita green space” has an obvious shortcoming and it cannot reflect the distribution structure and quality of green space [30]. However, combined with the “semi-closed” university campus, this shortcoming has been reduced. The higher the proportion of “Green space per capita”, the larger the green coverage area is. In this research, ArcGIS was used to label the green space in the school, which is divided by the school population.

*GSPC* = (*G*1 + *G*2 + *G*3) ÷ *NOS* (5)

*GSPC*: Green space per capita

*G*: Green space

*NOS*: Number of students

#### 2.2.4. Calculation Method of Walk Score

Walk score (WS) is a commercial walkability measurement tool based on the distance from a specific address to surrounding amenities. The fit index of the built environment used in this research is the community walking index proposed by [31] which can reflect the residents’ willingness to walk and the convenience in the process of walking. The higher the “Walk score” is, the more willing students are to choose walking and the more convenient the walking process becomes. As a special group, only a small number of students have electric vehicles or cars, so most of the students choose walking as their main travel mode, which also proves the feasibility of the formula in this study.

*WS* = (*6* × *LUM* × *Z*) + (*NPD* × *Z*) + (*ID* × *Z*) (6)

*WS*: Walk score

*LUM*: Land use mix

*NPD* (Net population density) = (Total population in school)/(Total school area)

*ID* (Intersection density) = (Number of school intersections)/(Total length of streets in school)

### 2.3. Measures of Physical Fitness

The indicators of physical fitness selected in this research all refer to Chinese students’ physical health standards (BMI, vital capacity, sit-and-reach, 800 m/1000 m running, 50 m running, pull-up, curl-up, grip strength).

The correlation between the built environment and physical activity has been proven by a large number of researchers [32,33,34,35,36,37,38,39]. Likewise, as the introduction mentioned, the benefits of physical activity to physical health are irrefutable, and virtually everyone can improve the quality of their lives by becoming more physically active [5,6].

### 2.4. Statistical Analysis

Statistical analyses were performed using SPSS version 26. For comparison of values, data distribution was analyzed for normality (Appendix A). Differences among 9 experimental groups were analyzed by Kruskal–Wallis test in an independent sample non-parametric test, followed by a pairwise comparison. The association between the built environment and students’ physical fitness was analyzed by Spearman correlation (Table 3). When the *p*-value was less than 0.05, it was considered to be correlational and significant.

## 3. Results

### 3.1. Physical Fitness of Students in Four Colleges

The difference in physical fitness of male students in four colleges is presented in Table 4. For vital capacity and grip strength, the grades of four colleges differs from each other. For sit-and-reach, the difference between S2 and S3, S1 and S2 was significant. For pull-ups, except for S3 and S2, the difference in others were all significant. For 50 m running, the difference in S3 and S1, S2, and S3 was significant. For 1000 m running, the difference was all significant, except for S2 and S3.

### 3.2. Built Environment of Four Colleges

The built environment of colleges is presented in Table 4. From the data, the built environment of Xi’an University of Architecture and Technology is better than the other three colleges on the whole. However, the green space per capita in Xi’an Jiao tong University is the largest of four schools.

The difference in physical fitness of female students in four colleges has been presented in Table 5. For grip strength, the grades of four colleges differ from each other, respectively, for vital capacity, and the difference was significant except for S1 and S4. For sit-and-reach the difference was significant except for S2 and S4, S1 and S3. For sit-up, the difference was significant except for S3 and S4. For 50 m running, the difference in S3 and S1, S2, and S3 was significant. For 800 m running, the difference was significant except for S3 and S4, S1 and S2.

### 3.3. The Correlation between the Built Environment and Students’ Physical Health

Because there are differences in test items between boys and girls, gender factors are controlled. It is known that there are significant differences in other physical fitness test items, except BMI. Specific results can be seen in Table 6.

After controlling the factors (school, gender, grade, and BMI), Spearman correlation was made on the built environment and students’ physical health, which came to the following results: For men, there was a significant correlation between street connectivity and vital capacity, grip strength, 50 m running, 1000 m running; a significant correlation between land use mix and vital capacity, sit-and-reach, pull-up, grip strength; a significant correlation between green space per capita and vital capacity, grip strength, 50 m running; and a significant correlation between walk score and vital capacity, pull-up, grip strength, 50 m running. For women, there was a significant correlation between street connectivity and vital capacity, grip strength, 50 m running, 800 m running, and curl-up; a significant correlation between land use mix and vital capacity, sit-and-reach, curl-up, grip strength, 800 m running; a significant correlation between green space per capita and vital capacity, grip strength, curl-up, sit-and-reach; and a significant correlation between walk score and vital capacity, curl-up, grip strength, 800 m running.

## 4. Discussion

Taken together, these results suggest that there was an association between campus-built environment and college students’ physical fitness, and that this correlation was different in gender. This result is consistent with our hypothesis. It is meaningful, as we could provide relevant government departments and schools with corresponding references for the future campus construction through deeper analysis.

There is little research that has studied the correlation between the built environment and physical fitness. A previous study examined this relationship in European adolescents where the authors accessed the built environment according to 5 factors (secure bicycle/walking route, heavy traffic in neighborhood, shops near home, outdoor fields). A negative association between heavy traffic and physical fitness was confirmed (*p* < 0.05). On the contrary, a secure bicycling or walking route from home to school and outdoor fields and gymnasiums near home was positively associated with physical fitness [12]. The limitations of this study were the lack of other factors of the built environment (street connectivity, land use mix, etc.), which were also important. Another study examined the relationship between adolescents’ perception of the architectural environment and aerobic fitness according to nine environmental factors (types of residences, stores, and other facilities in the neighborhood, access to services, street in the neighborhood, places for walking and cycling, neighborhood surroundings, safety from route and crime, and neighborhood satisfaction) [1]. However, the limitation of this study is that only aerobic fitness was included without the other components (muscular strength, flexibility, speed and agility, etc.), which are also important. In our study, four indicators of the built environment were all confirmed to be significantly associated with different indicators of physical fitness. These results differed from the research, which could be due to a discrepancy in the assessment of the built environment that was assessed subjectively in both studies. Additionally, the gaps of physical fitness should be considered, as Asian students’ physical fitness level was worse than that of European students [40].

Indeed, most indicators of physical fitness were influenced by the built environment in our study. However, there was barely any research that advanced the direct association between the built environment and physical fitness, as most of them explained this association as a “Mediation effect”—that the built environment could promote individuals to exercise more and then improve their physical fitness level.

In our study, SC, LUM, GSPC, and WS were all significantly positively correlated with students’ vital capacity and negatively correlated with 800 m/1000 m (the longer the time, the worse the grade). These results suggest that the built environment had a positive effect on college students’ physical fitness. Here are some studies have proved that street connectivity is related to the frequency of walking or cycling [35,37,39]. A positive correlation between land use mix within 800 m from home and people’s frequency of walking to stores was also confirmed [33,36]. In addition, with the increase of business types within 1600 m, the frequency of undirected walking and cycling traveling also increases [34]. Generally, this evidence together suggests that when people perceived accessibility to facilities for daily necessities use, they were more willing to walk and bicycle, which promotes their physical activity directly [41]. Thirdly, there are mounting studies that confirm that green space can provide more opportunities for sports activities, relieve stress and attention fatigue, and promote social contact. Correspondingly, the correlation between green space and physical activity has been proven in many countries, such as China [42], England [6], Australia [43], the USA [44], etc. Finally, the higher the walk score is, the more willing people are to choose walking [45]. The main physical activities of students in college include Work (Study)-related PA, Leisure time PA, and Transportation-related PA. Therefore, a favorable campus environment (GSPC), convenient and safe design of streets (SC), and sensible building layout (LUM) may encourage students to choose walking as the main travel option, which could affect their physical fitness positively.

The positive correlation between grip strength and built environment was another significant result of our study. Grip strength was highly correlated with other physical fitness indicators within all ages, which has gained much attention as a sensitive and independent indicator of physical fitness [46]. A study published in The Lancet showed that a 5 kg reduction in grip strength was associated with a 16% increase in the risk of death, a 7% increase in the risk of heart attack, and a 9% increase in the risk of stroke [47]. Our results may confirm previous findings—that grip strength may indirectly be influenced by the built environment.

In our study, female students’ grade of sit-and-reach was positively associated with SC, LUM, and GSPC, which was not found among male students. The gaps in growth may be the reason for this result. To male students, the growth of strength was better than female students, while for female students, their growth of flexibility was better. Our results showed that the effect of the built environment to 50 m running was more significant in male students. On the contrary, the effect of the built environment to 800 m/1000 m running was more significant in female students. Both of the two test items could reflect the muscular endurance, 50 m running required the anaerobic capacity, while 800 m/1000 m running required aerobic capacity. This may prove that gender may be one of the factors affecting the impact of the built environment on physical fitness. Additionally, many other studies also concluded that growth is the most relevant factor in teenagers’ physical fitness [43,48].

Combining the results and discussion above, the results of our study could be proven. Here are some suggestions for the construction of a campus-built environment:

1. While designing the road of the college, the number of short links and intersections deserves to be increased. It can not only slow down the speed of drivers to reduce the incidence of traffic accidents, but also increase the route selections for students. However, street connectivity has different effects on community networks of different sizes and types. In other words, the street connectivity that is applicable to one network type is not always applicable to other types.
2. According to the site selection of the buildings in college, as the most common place for students to go, the distance between the student dormitory and academic building should be suitable. Too far will reduce the probability of walking for students, while too short might not satisfy the requirement of physical activity. Furthermore, the types of sports venues should be as many as possible, and the distance should not be too far from the dormitory.

One of the advantages of this study is selecting college students as subjects. As mentioned before, college students are in a special period (late adolescence and early adulthood), and the attitudes towards and knowledge of health formed at this time may continue throughout their life. Since the correlation between the built environment and physical fitness has been proved, the necessity of improving the campus-built environment is also increasing. Another advantage is the new findings on the correlation between muscular strength, speed, and agility with the built environment, which could add new information to the literature.

However, the reader should bear in mind that the study is based on physical fitness and built environment, which ignores the physical activities. Another limitation is that ArcGIS is the only means of measurement, while some indicators of the built environment were more suitable to be measured through field investigation. Finally, the number of colleges was small, and it would be better if the indicators of built environment in students were more adequate.

## 5. Conclusions

The results suggest that there is a correlation between campus-built environment and college students’ aerobic capacity, muscle strength, and speed agility qualities. Gender may play an important role in this correlation. The vital capacity and grip strength both had a significant correlation with four factors of built environment in our study, which could add new information to the literature. Therefore, we suggest that while designing the road of the college, the number of short links and intersections deserves to be increased. Furthermore, the location of student dormitories and academic buildings should be suitable. Around 0.5–1.7 km may be an appropriate distance between student dormitories and academic buildings.

## Figures and Tables

**Table 1:** Sample characteristics (*n* = 1498).

	S1	S2	S3	S4	Total
**Gender**					
**Men (%)**	187 (45.3%)	192 (49.1%)	197 (47.9%)	154 (54.4%)	730 (48.7%)
**Women**	226 (54.7%)	199 (50.9%)	214 (52.1%)	129 (45.6%)	768 (51.3%)
**Grade**					
**Freshman year**	110 (26.6%)	103 (26.4%)	6 (1.5%)	0 (0.0%)	
**Sophomore year**	105 (25.4%)	95 (24.3%)	79 (19.2%)	107 (37.8%)	
**Junior year**	113 (27.4%)	94 (24.0%)	83 (20.2%)	97 (34.3%)	
**Senior year**	85 (20.6%)	99 (25.3%)	243 (59.1%)	79 (27.9%)	
**Total**	413	391	411	283	1498

**Table 3:** The results of Kruskal–Wallis test: the difference in physical fitness of male students.

	S1	S2	S3	S4	*p*
**BMI**	22.31 ± 3.09	22.25 ± 2.90	22.12 ± 2.67	21.87 ± 2.47	0.556
**Vital capacity**	4106 ± 697	4615 ± 899	4338 ± 666	3997 ± 648	<0.01
**Sit-and-reach**	12.00 ± 6.41	13.71 ± 6.18	10.99 ± 6.09	12.78 ± 6.12	<0.01
**Pull-up**	3.28 ± 3.44	4.96 ± 4.40	5.51 ± 5.75	6.77 ± 5.57	<0.01
**Grip strength**	40.84 ± 19.21	39.47 ± 14.25	41.95 ± 21.85	38.54 ± 19.32	<0.01
**50 m running**	7.77 ± 0.63	7.82 ± 0.66	7.56 ± 0.52	7.71 ± 0.55	<0.01
**1000 running**	267.57 ± 32.00	262.92 ± 26.50	257.54 ± 26.51	248.95 ± 29.14	<0.01

**Table 4:** Summary of the built environment of four colleges (*n* = 4).

	S1	S2	S3	S4
**Street connectivity**	0.87	0.93	0.90	0.96
**Land use mix**	24.18%	34.02%	6.77%	11.78%
**Green space per capita**	25.29%	43.03%	49.24%	25.91%
**Walk score**	70	82	78	73

**Table 5:** The results of Kruskal–Wallis test: the difference in physical fitness of female students.

	S1	S2	S3	S4	*p*
**BMI**	21.17 ± 2.65	20.77 ± 2.53	21.21 ± 2.50	20.67 ± 2.36	0.064
**Vital capacity**	2592 ± 477	3013 ± 559	2855 ± 481	2778 ± 516	<0.01
**Sit-and-reach**	15.25 ± 6.53	17.35 ± 5.20	14.95 ± 6.15	17.20 ± 5.27	<0.01
**Sit-up**	26.99 ± 9.20	36.38 ± 6.80	31.81 ± 9.93	31.00 ± 7.99	<0.01
**Grip strength**	25.20 ± 13.10	26.21 ± 12.35	27.25 ± 15.60	24.32 ± 12.69	<0.01
**50 m running**	9.83 ± 0.87	9.88 ± 0.81	9.48 ± 0.76	9.62 ± 0.70	<0.01
**800 running**	263.73 ± 24.38	265.37 ± 24.99	254.07 ± 26.24	252.78 ± 27.21	<0.01

**Table 6:** The results of Spearman correlation: associations of students’ physical health with the built environment in school.

	Mean ± SD (Male)	SC (*p*)	LUM (*p*)	GSPC (*p*)	WS (*p*)
**BMI**	22.14 ± 2.81	-	-	-	-
**Vital capacity**	4280.16 ± 774.09	0.011	<0.01	<0.01	<0.01
**Sit-and-reach**	12.34 ± 6.28	0.308	0.465	0.975	0.812
**Pull-up**	5.08 ± 5.00	0.523	<0.01	0.101	0.003
**Grip strength**	41.95 ± 21.85	0.012	0.014	0.096	<0.01
**50 m running**	7.71 ± 0.60	0.154	0.112	<0.01	0.017
**1000 running**	259.71 ± 29.27	0.124	0.251	0.128	<0.01
	**Mean ± SD (Female)**				
**BMI**	21.00 ± 2.54	-	-	-	-
**Vital capacity**	2778.91 ± 516.51	0.003	0.003	<0.001	<0.001
**Sit-and-reach**	16.04 ± 5.99	0.283	<0.001	<0.001	0.017
**Curl-up**	31.44 ± 9.33	<0.001	0.145	0.125	<0.001
**Grip strength**	25.20 ± 13.10	<0.001	0.245	<0.001	<0.001
**50 m running**	9.71 ± 0.823	0.676	0.672	0.199	0.340
**800 running**	259.63 ± 26.0	0.002	0.002	0.957	0.032

**Figure A1:**
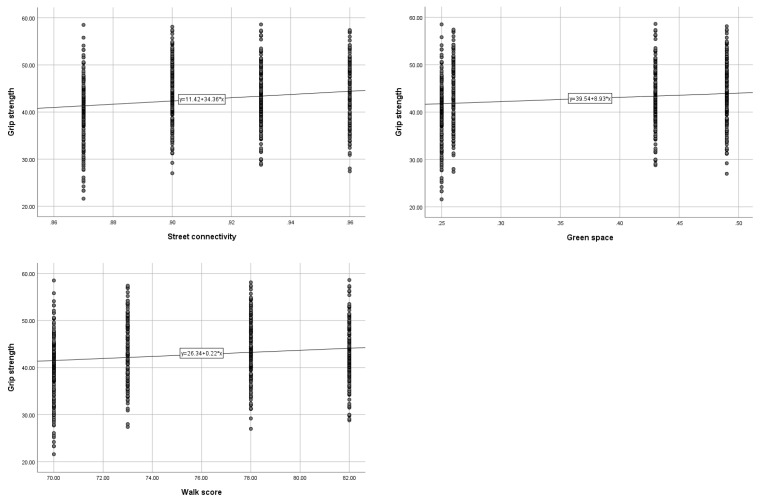
The specimen scatterplots for correlation between grip strength and built environment in male students.

**Figure A2:**
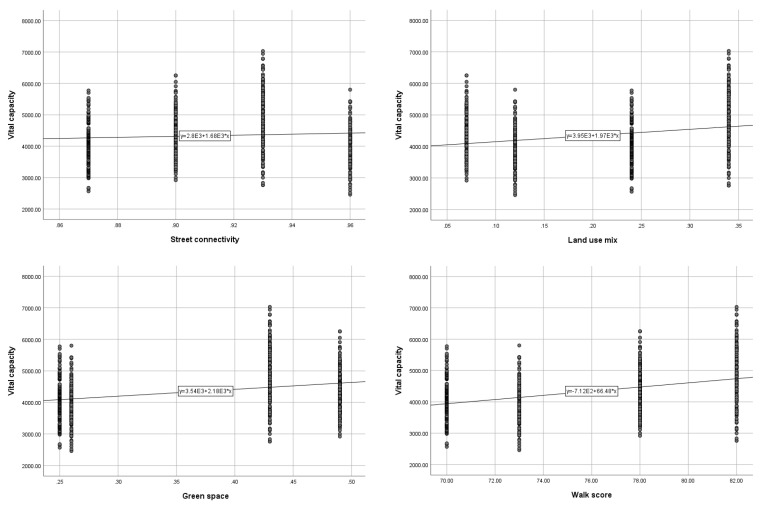
The specimen scatterplots for correlation between vital capacity and built environment in male students.

**Figure A3:**
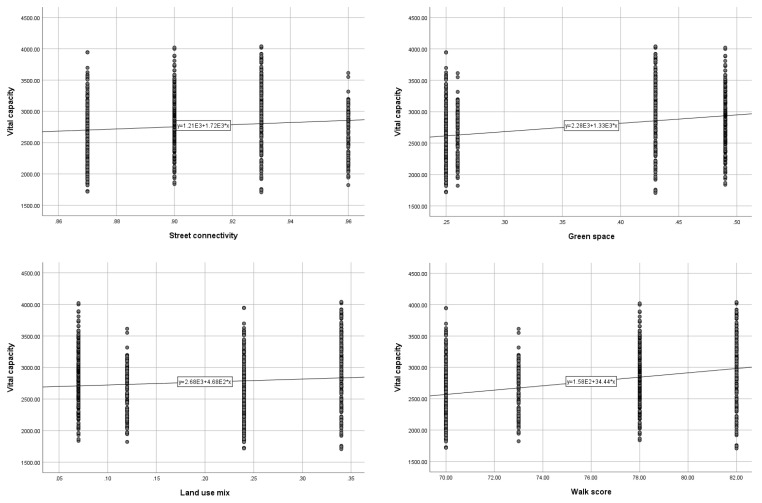
The specimen scatterplots for correlation between vital capacity and built environment in female students.

**Figure A4:**
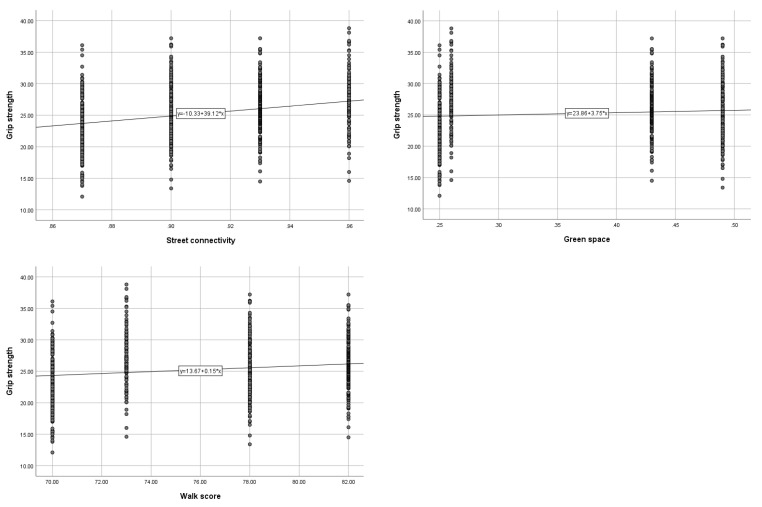
The specimen scatterplots for correlation between grip strength and built environment in female students.

## Data Availability

Not applicable.
